# Using Ecological Momentary Assessment to Test the Effectiveness of a Web-Based Brief Alcohol Intervention Over Time Among Heavy-Drinking Students: Randomized Controlled Trial

**DOI:** 10.2196/jmir.2817

**Published:** 2014-01-08

**Authors:** Carmen Voogt, Emmanuel Kuntsche, Marloes Kleinjan, Evelien Poelen, Rutger Engels

**Affiliations:** ^1^Behavioural Science InstituteRadboud University NijmegenNijmegenNetherlands; ^2^Addiction Switzerland, Research InstituteLausanneSwitzerland

**Keywords:** intervention study, drinking, students

## Abstract

**Background:**

Web-based brief alcohol interventions are effective in reducing alcohol use among students when measured at limited follow-up time points. To date, no studies have tested Web-based brief alcohol intervention effectiveness over time by using a large number of measurements.

**Objective:**

Testing whether the What Do You Drink (WDYD) Web-based brief alcohol intervention can sustain a reduction in alcohol use among heavy-drinking students aged 18-24 years at 1-, 3-, and 6-month follow-up intervals.

**Methods:**

A purely Web-based, 2-arm, parallel-group randomized controlled trial applying an ecological momentary assessment approach with 30 weekly measurements was conducted in the Netherlands (2010-2011). Participants were recruited offline and online. A total of 907 participants were randomized into the experimental condition (n=456) including the single-session and fully automated WDYD intervention, or into the control condition (n=451) including assessment only. Weekly alcohol consumption and frequency of binge drinking were the self-assessed outcome measures.

**Results:**

Attrition rates of the 907 participants were 110 (12.1%), 130 (14.3%), and 162 (17.9%) at 1-, 3-, and 6-month follow-up intervals, respectively. Latent growth curve analyses according to the intention-to-treat principle revealed that participants in the experimental condition had significantly lower weekly alcohol consumption compared to participants in the control condition that was sustained at 3-month follow-up (intercept=–2.60, *P*<.001; slope=0.16, *P*=.08). Additional linear regression analyses indicated that this intercept difference resulted from significantly higher levels of alcohol units per week for participants in the control condition compared to those in the experimental condition at 1-month (beta=–2.56, SE 0.74, Cohen’s *d*=0.20, *P*=.001), 3-month (beta=–1.76, SE 0.60, Cohen’s *d*=0.13, *P*=.003), and 6-month (beta=–1.21, SE 0.58, Cohen’s *d*=0.09, *P*=.04) follow-up intervals. Latent growth curve analyses further indicated that participants in the experimental condition had a significantly lower frequency of binge drinking compared to participants in the control condition that was sustained at 6-month follow-up (intercept=–0.14, *P*=.01; slope=0.004, *P*=.19). This intercept difference resulted from higher levels in this outcome for participants in the control condition relative to participants in the experimental condition at 1-month (beta=–1.15, SE 0.06, Cohen’s *d*=0.16, *P*=.01), 3-month (beta=–0.12, SE 0.05, Cohen’s *d*=0.09, *P*=.01), and 6-month (beta=–0.09, SE 0.05, Cohen’s *d*=0.03, *P*=.045) follow-up intervals.

**Conclusions:**

The WDYD intervention was shown to be effective in preventing an increase in weekly alcohol consumption and frequency of binge drinking directly after the intervention. This effect was sustained 3 and 6 months after the intervention.

**Trial Registration:**

Netherlands Trial Register NTR2665; http://www.trialregister.nl/trialreg/admin/rctview.asp?TC=2665 (Archived by WebCite at http://webcitation.org/6LuQVn12M).

## Introduction

Given the high prevalence and social and economic costs attributable to heavy drinking among young adults, there is an urgent need for adequate interventions [1-3]. The widespread growth and availability of computer technology and the Internet has provided the opportunity to deliver interventions via the Web [4], which is advantageous for young adults because it allows them to access information at a self-selected time and place while remaining anonymous [5]. Web-based brief alcohol interventions have been found to be effective in reducing the quantity and frequency of alcohol use among heavy-drinking young adults and students [6-9]. However, despite the demonstrated effectiveness of these types of interventions, findings have to be interpreted with caution because of the way alcohol use and intervention effectiveness are assessed. First, alcohol use is typically assessed over relatively long recall periods (eg, 30 days) and participants are often asked to report the average number of alcohol units they consumed in a usual week. This can result in measurement errors because precise recall of alcohol use decreases after 2 or 3 days due to memory deficits [10]. Second, the fluctuating nature of alcohol use among students because of calendar-specific events [1,11] is often overlooked because intervention effectiveness is measured at limited follow-up time points (ie, 1 short and several longer follow-ups of approximately 1-12 months after the intervention). The use of limited follow-up time points not only disregards important drinking events, but also increases the danger of drawing inaccurate conclusions about intervention effectiveness. In a previous study, we recognized the disadvantage of using only 2 follow-up time points (ie, 1 and 6 months) when testing the effectiveness of the What Do You Drink (WDYD) Web-based brief alcohol intervention. Our baseline assessment was completed during a usual drinking period without any remarkable events, whereas our first 1-month follow-up assessment coincided with carnival, a 4-day event associated with excessive drinking. The observed increase in alcohol use was likely the consequence of the selection of this particular week to test intervention effectiveness rather than the WDYD intervention, which aims to detect and reduce heavy drinking in young adults [12]. The single-session and fully automated WDYD intervention was developed in collaboration with the Trimbos Institute (Netherlands Institute of Mental Health and Addiction) by using the intervention mapping protocol [13]. Content is based on motivational interviewing principles [14] and parts of the I-Change model [15] in which knowledge, social norms, and self-efficacy are embedded as the most changeable determinants of behavior change [12]. Despite the sound theoretical background of the WDYD intervention, no significant main effects on alcohol use were found when using 2 follow-up time points with short recall periods of 7 days [5]. Third, individual changes in alcohol use and intervention effectiveness over time remain unnoticed when using limited follow-up time points. Information on these changes can be extremely valuable for determining the time at which the intervention effects have stopped and the time at which booster sessions may be needed to strengthen and/or extend intervention effects.

In evaluating intervention effectiveness, it appears to be important to use short recall periods to reduce measurement errors and to include a large number of measurements to consider the fluctuating nature of alcohol use over time and capture important drinking events. Higher precision in establishing intervention effectiveness can be achieved by means of ecological momentary assessment (EMA) and latent growth curve (LGC) modeling techniques. The ecological aspect of EMA is that data are collected in real-life settings at strategically selected moments in time [16]. The momentary aspect of EMA implies that the assessment of alcohol use focuses on participants’ current or recent state. In addition, EMA is characterized by repeated and a large number of measurements over time and often used equivalent to experience sampling methods (ESM), a systematic way for participants to report on their ongoing alcohol use behavior [17,18]. LGC modeling techniques allow for estimation of average growth trajectories (ie, mean intercepts and slopes) of alcohol use over time as well as individual differences in these trajectories (ie, intercept and slope variances) [19,20]. The estimation of variances in growth trajectories increases the reliability of outcome measures. This is not possible with traditional statistical techniques that are often used to test intervention effectiveness, such as repeated-measures ANOVA [21], because they only provide mean growth patterns and treat variances as error [22]. Although the advantages of employing EMA and LGC modeling techniques are evident, most trials on Web-based brief alcohol interventions used long recall periods with limited follow-up time points and traditional techniques [21] to test intervention effectiveness [4]. To our knowledge, this is the first study to test whether a Web-based brief alcohol intervention can sustain a reduction in alcohol use among heavy-drinking students at 1-, 3-, and 6-month follow-up intervals. To test the effectiveness of the WDYD intervention over time, we conducted 30 weekly EMA measurements for 6 months through online surveys and LGC analyses to model individual change in weekly alcohol consumption and frequency of binge drinking at 1-, 3-, and 6-month follow-up intervals by condition. The rationale of reporting over 3 time periods was to gain insight into how long the intervention effects sustained and to limit the chance of reporting outlier trajectories. We hypothesized that participants in the experimental condition would reduce their alcohol use (intercept) compared to participants in the control condition directly after exposure to the WDYD intervention. Based on Web-based brief alcohol interventions that have produced long-term effects [8,9], it was hypothesized that the reduction would be sustained (slope) at the 6-month follow-up interval.

## Methods

### Study Design

A 2-arm, parallel-group randomized controlled trial applying an EMA approach with 30 weekly EMA measurements was conducted online in the Netherlands (2010-2011) to test whether the WDYD intervention could sustain a reduction in alcohol use among heavy-drinking students at 1-, 3-, and 6-month follow-up intervals. This trial was purely Web-based because there were no face-to-face components in the intervention and for assessing the outcome measures.

### Procedure and Participants

A convenience sampling strategy was used to recruit heavy-drinking students offline by distributing flyers at universities and higher professional education institutions (ie, universities of applied sciences) online by sending emails with information about the study from September to December 2010. The cover story was that students had to evaluate newly developed health education materials addressing alcohol use and that they had to judge these materials to reduce the risk of social desirability bias. Students were blinded to the aim of the study until the end of the EMA study. Interested students were referred to an email address and were sent a detailed description of the study by email. To be included in the study, students had to (1) be between ages 18 and 24 years, (2) report heavy drinking in the past 6 months, (3) be ready to change their alcohol use, (4) have daily access to the Internet (and be literate), and (5) sign an online informed consent form. Heavy drinking was defined as consuming more than 14 (females) or 21 (males) glasses of standard alcohol units per week and/or consuming 5 or more glasses of standard alcohol units per occasion at least 1 day per week [23]. Students reporting a score of 20 or higher on the Alcohol Use Disorders Identification Test (AUDIT) [24], and/or receiving treatment for alcohol-related problems were excluded from the study and advised to seek treatment because the WDYD intervention was developed to reduce heavy drinking rather than problem drinking.

A sample size of 908 participants was necessary to detect an increase in the percentage of participants showing low-risk drinking guidelines after 1 month of 42% in the experimental condition versus 31% in the control condition [25] with a 2-sided 5% significance level and a power of 80%, given an anticipated dropout rate of 30% after randomization. Students who met the inclusion criteria were randomly assigned to the WDYD intervention condition (n=456) or to the control condition (n=451) in blocks of 4 using a computerized random number generator by an independent researcher of the Behavioural Science Institute who could not influence or predict the randomization result. Participants were not blinded to randomization results. Randomization was stratified by sex and educational level before the baseline assessment in January 2011 [23].

In total, 30 weekly EMA measurements were conducted online from January to August 2011, to assess outcome measures with 4 pretests and 26 posttests. After the 4 pretests in January, participants in the experimental condition were exposed to the WDYD intervention, whereas those in the control condition received assessment only. Directly after intervention exposure in the first week of February, participants in both conditions received the first posttest. One week after the intervention, all participants received weekly EMA posttest measurements for 6 months from February to August. EMA measurements were assessed on Monday mornings. All participants received an email with the instructions on the use of the survey, and they were asked to respond to the survey before midnight. Each survey took approximately 10 minutes to complete and contained identical questions about participants’ weekly alcohol consumption, frequency of binge drinking, and drinking refusal self-efficacy. In addition, extended surveys were administered at baseline assessment, immediately after the intervention, and at 1 and 6 months after the intervention. These extended surveys included additional questions concerning alcohol-related cognitions, cost-effectiveness, and problem drinking. Completion time of the extended surveys was approximately 20 minutes. Paper-and-pencil surveys with identical content were provided to participants in case they were unable to access the Internet. Participants who failed to complete the survey on Mondays received a short text message on their mobile phones on Tuesdays to remind them. Those who still did not complete the survey on Tuesdays were reminded by a telephone call on Wednesdays. On average, 11% (range 7%-17%) of the surveys were completed on Tuesdays and Wednesdays instead of Mondays. When participants completed at least 28 of 30 surveys, they received €100 as an incentive, as stated in the informed consent. Ethical approval was provided by the Ethical Committee of the Faculty of Social Sciences at Radboud University Nijmegen (ECG30062011). This trial is registered at the Netherlands Trial Register (NTR2665) as mentioned in the trial protocol [23].

### Interventions

**Figure 1:**
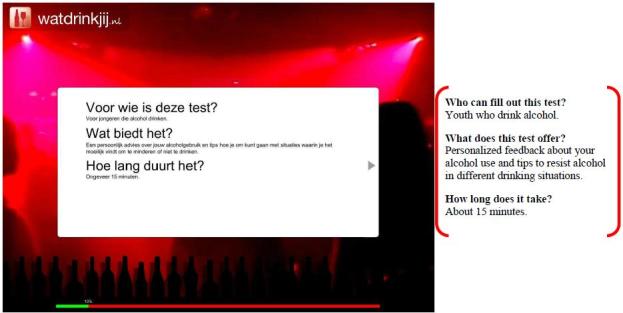
Screenshot and English translation of the What Do You Drink home page.

**Figure 2:**
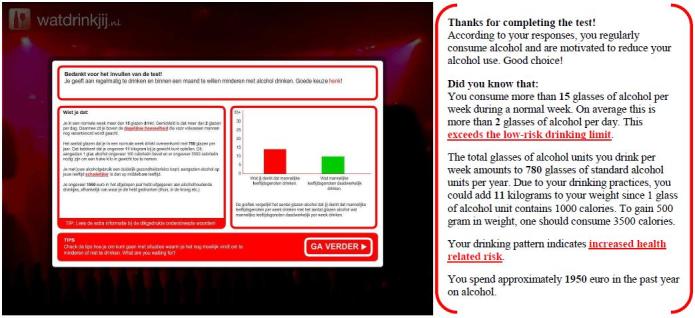
Screenshot and English translation of personalized feedback on the What Do You Drink website.

**Figure 3:**
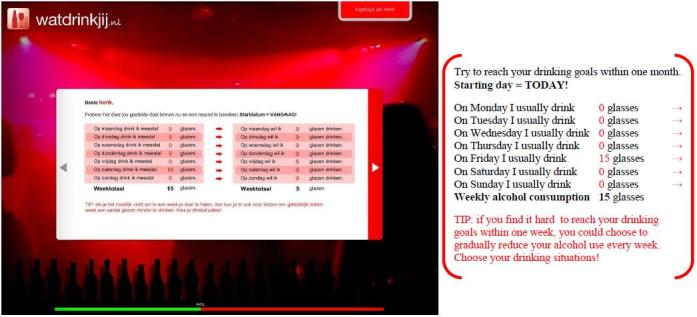
Screenshot and English translation of drinking goals on the What Do You Drink website.

**Figure 4:**
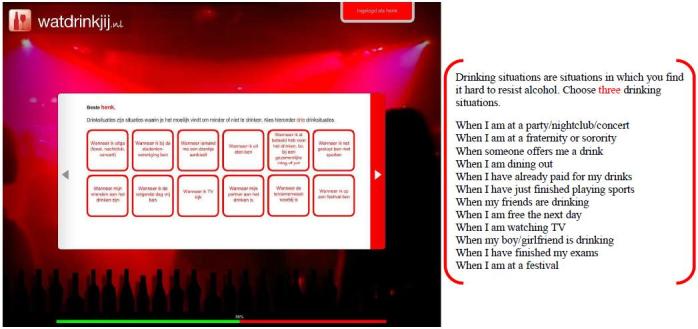
Screenshot and English translation of the overview of drinking situations on the What Do You Drink website.

Participants assigned to the experimental condition used the WDYD intervention. The first part of the WDYD intervention focuses on increasing the users’ awareness of the potential problems, consequences, and risks associated with their drinking behavior. It contains a home page and a screening test with personalized feedback delivered in a nonjudgmental, nonconfrontational, and nonaversive way (see Figure 1). The screening test included participants’ self-reported name, sex, age, education level, weight, alcohol use, readiness to change alcohol use, average expenses for consumed alcohol beverages, and descriptive social norms. Personalized feedback consisted of advice about drinking according to low-risk drinking guidelines [26], personal drinking profile (quantity-frequency consumed in past year), estimates of calorie intake, increases in weight, money expenses because of drinking, and a comparison of personal use rates with the national norms of same-sex peers to correct misperceptions of descriptive social norms (see Figure 2). The personalized feedback was based on the individuals’ personal situation, implying that the WDYD intervention was tailored. The second part of WDYD focused on setting and maintaining drinking goals (see Figure 3) and strengthening users’ drinking refusal self-efficacy to succeed and maintain drinking goals by providing tips to resist alcohol in different drinking situations (see Figure 4). Participants were able to track their progress through the WDYD intervention, which took approximately 20 minutes to complete. A full description of the WDYD intervention is given elsewhere [12]. Participants assigned to the control condition received assessment only.

### Outcome Measures

Weekly alcohol consumption and frequency of binge drinking were the self-assessed primary outcome measures through online surveys. Weekly EMA measurements were used to assess the primary outcome measures over time at 1-, 3-, and 6-month follow-up intervals.

Weekly alcohol consumption, defined as the mean number of glasses of standard alcohol units consumed in the previous 7 days, was assessed using the Dutch version of the Alcohol Weekly Recall [27]. Participants were asked to indicate retrospectively the exact number, size, and type of alcohol beverage they consumed on each day of the previous 7 days. An overview of standard units for various beverages was provided to guarantee standardized responses. In total, 1.47% of the participants scored above 3 standard deviations of the sample mean of weekly alcohol consumption, but they were given a score exactly at 3 standard deviations above the sample mean of weekly alcohol consumption to retain outliers in the analyses (resulting range 0-109) [28] and to handle outliers in accordance with our previous studies [5,29]. Binge drinking frequency, defined as the number of days in the previous week on which participants drank 5 or more glasses of standard alcohol units per occasion [5], was assessed on an 8-point Likert scale ranging from 0=never to 7=every day.

### Analyses

Data were analyzed according the intent-to-treat (ITT) principle. Missing data were handled by using multiple imputations using the predictive mean matching method [5,30]. Twenty imputed datasets were evaluated with *P*<.05 as the criterion for statistical significance by averaging the results (ie, pooling). First, descriptive analyses involving *t* tests and chi-square tests were conducted to explore whether the randomization resulted in a balanced distribution of participants’ demographic characteristics and alcohol use (ie, weekly alcohol consumption and frequency of binge drinking) across conditions at baseline assessment. Loss to follow-up was also examined with attrition analyses using 1-, 3-, and 6-month follow-up intervals as outcome measures and demographic characteristics, alcohol use, and condition status (intervention vs control) as predictors. Second, LGC analyses were conducted to model individual change in alcohol use over time by condition at 1-, 3-, and 6-month follow-up intervals. LGC analyses were conducted over 3 time periods to limit the chance of reporting outlier trajectories. The models without condition status and baseline levels of alcohol use were tested first. Subsequently, the growth curves were regressed on condition status for weekly alcohol consumption and frequency of binge drinking separately while adjusting for baseline levels of alcohol use. A random-effect parameter for educational institutions was not included in the models since variation in participants between institutions was expected to be limited because all participants needed to meet the inclusion criteria of the study.

Unstandardized intercepts, representing alcohol use directly after the intervention, and unstandardized slopes, representing the change of alcohol use over time, were reported. Global fit indexes were used to assess model fit for each construct: chi-square statistic, Comparative Fit Index (CFI), Tucker-Lewis Index (TLI) with a cut-off value of ≥0.90 and ≥0.95 for acceptable fit, and Root Mean Square Error of Approximation (RMSEA) with a cut-off value of ≤0.06 for acceptable fit [31]. In parallel with the LGC analyses, linear regression analyses were conducted for weekly alcohol consumption and frequency of binge drinking at 1-, 3-, and 6-month follow-up intervals and presented as unadjusted and adjusted for baseline levels of alcohol use to provide additional specific tests of a difference between the conditions. For the linear regression analyses, unstandardized coefficients (betas), standard errors (SE), and Cohen’s *d* [32] effect sizes were provided. All analyses were performed using Mplus version 6.0 [20].

In total, 30 weekly EMA measurements were conducted with 4 pretests and 26 posttests. For the LGC and linear regression analyses, the 4 pretests were aggregated into a baseline score. The first posttest immediately after the intervention was excluded from both analyses because participants reported on outcome measures over the previous week, thereby making it impossible to observe direct intervention effects. Thus, for all analyses, 4 pretests and 25 posttests were used. Only for the additional linear regression analyses, aggregated scores were computed for the 1-month follow-up interval (posttests 1-4), the 3-month follow-up interval (posttests 1-12), and the 6-month follow-up interval (posttests 1-25).

## Results

### Participant Flow

**Figure 5:**
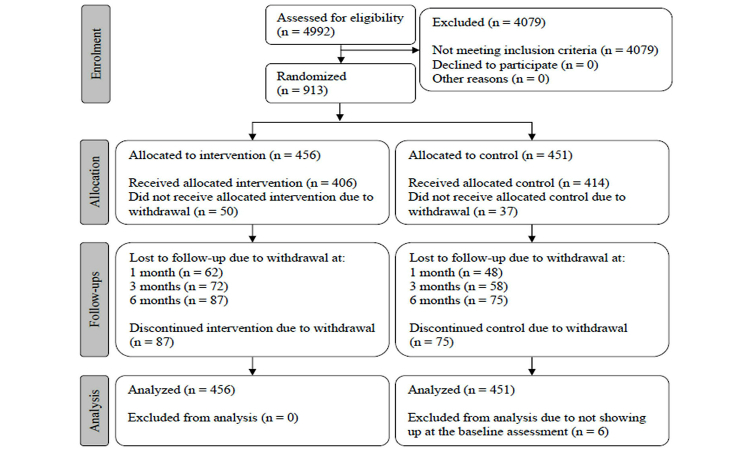
Participant flow diagram.

Figure 5 illustrates the participant flow through the study following the Consolidated Standards of Reporting Trials (CONSORT) guidelines [33] and the data collection with 30 weekly EMA measurements. Originally, 913 students were included in the study. However, 6 students did not fill in the baseline assessment and were excluded from the study. Finally, 907 participants were enrolled in the EMA study, randomized into the experimental condition (n=456, 50.3%) or control condition (n=451, 49.7%), and eligible for the ITT analyses. In total, 82.1% (745/907) completed the baseline assessment and all 25 EMA follow-ups.

### Descriptive Statistics

Of the 907 participants, 547 (60.3%) were male, 667 (73.5%) attended university, and 194 (21.4%) were ready to reduce alcohol use in the near future at baseline assessment. The screening survey was administered between September and December 2010, whereas the baseline assessment was administered in January 2011, which might explain the lower rates of participant’s readiness to change alcohol use at baseline assessment. On average, participants were age 20.8 years (SD 1.7). At baseline assessment, participants reported to consume a mean 21.9 (SD 13.5) alcohol units per week and reported to have 1.8 (SD 1.0) occasions in which they drank 5 or more glasses of alcohol units per week (see Table 1). No significant differences emerged between conditions in demographic characteristics and outcome measures at baseline assessment (analyses not shown here).

**Table 1:** Demographic characteristics and outcome measures at baseline assessment.

Demographic characteristics	Intervention (n=456)	Control (n=451)	Total sample (N=907)
Male, n (%)	275 (60.3)	272 (60.3)	547 (60.3)
Age, mean (SD)	20.9 (1.7)	20.8 (1.7)	20.8 (1.7)
Higher professional education, n (%)	122 (26.8)	118 (26.2)	240 (26.5)
University education, n (%)	334 (73.2)	333 (73.8)	667 (73.5)
Contemplation stage^a^, n (%)	93 (20.4)	101 (22.4)	194 (21.4)
Weekly alcohol consumption, mean (SD)	22.2 (12.9)	22.1 (13.8)	21.9 (13.5)
Frequency of binge drinking, mean (SD)	1.8 (1.0)	1.7 (1.1)	1.8 (1.0)

^a^Readiness to change alcohol use was assessed through a question asking the participants which statement applied best to them. Participants selecting “I want to reduce drinking alcohol within the upcoming 6 months” or “I want to reduce drinking alcohol within the upcoming month” were considered to be in the contemplation stage of change, meaning that they were willing to reduce their alcohol use in the near future.

### Loss to Follow-Up

Because of lack of interest and time, the attrition rate at the 1-month follow-up interval was 12.1% (110/907; intervention: 62/456, 13.6%; control: 48/451, 10.6%), 14.3% at the 3-month follow-up interval (130/907; intervention: 72/456, 15.8%; control: 58/451, 12.9%), and 17.9% at 6-month follow-up interval (162/907; intervention: 87/456, 19.1%; control: 75/451, 16.6%). Attrition was not related to conditions at 1-, 3-, and 6-month follow-up intervals (χ^2^_1_=1.9, *P*=.17; χ^2^_1_=1.6, *P*=.21; and χ^2^_1_=0.9, *P*=.34). Completers (those who completed the baseline assessment and all 25 EMA follow-ups, n=745) did not differ from noncompleters (n=162) with respect to demographic characteristics (ie, sex: χ^2^_1_=0.3, *P*=.56; age: *t*_902_=–0.25, *P*=.80; education: χ^2^_1_=1.9, *P*=.17; and readiness to change alcohol use: χ^2^_1_=0.1, *P*=.73), and alcohol use (ie, weekly alcohol consumption: *t*_903_=0.32, *P*=.75; frequency of binge drinking: *t*_903_=–0.57, *P*=.57) at baseline assessment. The distribution of the missing values indicated that 87 of 907 participants (9.6%) did not complete the EMA study and that 75 of 907 participants (8.3%) nearly completed the survey (missing 1 or 2 of 30 EMA measurements).

### Model Findings

The models for weekly alcohol consumption and frequency of binge drinking without condition status and baseline levels of alcohol use were tested first. The intercept and slope of weekly alcohol consumption were significant (intercept=23.7, *P*<.001; slope=–0.06, *P*=.002), indicating that participants consumed 23.7 alcohol units on average and gradually reduced their consumption throughout the 6-month study period (χ^2^_320_=1393.2, *P*<.001; CFI=0.90; TLI=0.91; RMSEA=0.06). For frequency of binge drinking, a significant intercept and slope was found (intercept=1.9, *P*<.001; slope=–0.01, *P*<.001), meaning that the average number of occasions in the previous week that participants had drunk 5 or more glasses of alcohol units was 1.89. Participants’ frequency of binge drinking slowly reduced throughout the 6-month study period (χ^2^_320_=904.2, *P*<.001; CFI=0.92; TLI=0.92; RMSEA=0.05). Next, condition status and baseline levels of alcohol were added to both models. The weekly alcohol consumption model provided an acceptable fit to the data at follow-up assessments, except for the RMSEA at the 1-month follow-up interval. Fit indexes for weekly alcohol consumption were χ^2^_9_=107.3, *P*<.001, CFI=0.95, TLI=0.92, and RMSEA=0.11 at the 1-month follow-up interval, χ^2^_93_=644.7, *P*<.001, CFI=0.91, TLI=0.91, and RMSEA=0.08 at the 3-month follow-up interval, and χ^2^_366_=1451.2, *P*<.001, CFI=0.91, TLI=0.91, and RMSEA=0.06 at the 6-month follow-up interval.

**Figure 6:**
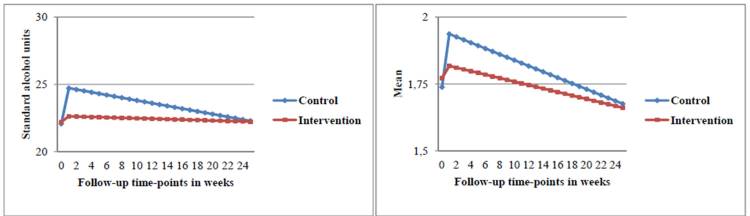
Left: latent growth trajectory for weekly alcohol consumption by condition after 6 months follow-up. Right: latent growth trajectory for frequency of binge drinking by condition after 6 months follow-up.

The LGC analyses revealed that participants in the experimental condition had a significantly lower weekly alcohol consumption compared to participants in the control condition directly after the intervention. The intercept difference in alcohol units between conditions sustained at the 3-month follow-up interval (intercept=–2.60, *P*<.001; slope=0.16, *P*=.08), but faded out over time resulting in a significant slope of the LCG at the 6-month follow-up interval (intercept=–2.18, *P*=.001; slope=0.08, *P*=.02) (see Table 2 and Figure 6). Linear regression analyses indicated that the intercept difference resulted from significantly higher levels of alcohol units per week for participants in the control condition compared to those in the experimental condition at 1-month (beta=–2.56; SE 0.74; Cohen’s *d*=0.20; *P*=.001), 3-month (beta=–1.76; SE 0.60; Cohen’s *d*=0.13; *P*=.003), and 6-month (beta=–1.21; SE 0.58; Cohen’s *d*=0.09; *P*=.04) follow-up intervals (see Table 3).

The frequency of binge drinking model provided an acceptable fit at all 3 follow-up intervals. Fit indexes for frequency of binge drinking were χ^2^_9_=42.3, *P*<.001, CFI=0.97, TLI=0.95, and RMSEA=0.06 at the 1-month follow-up interval, χ^2^_93_=341.3, *P*<.001, CFI=0.93, TLI=0.93, and RMSEA=0.05 at the 3-month follow-up interval, and χ^2^_366_=956.9, *P*<.001, CFI=0.92, TLI=0.93, and RMSEA=0.04 at the 6-month follow-up interval. According to the LGC analyses, the frequency of binge drinking of participants in the experimental condition was significantly lower compared to participants in the control condition. The intercept difference in frequency of binge drinking was sustained at the 6-month follow-up interval (intercept=–0.14, *P*=.01; slope=0.004, *P*=.19) (see Table 2) and resulted from higher levels in this outcome for participants in the control condition relative to participants in the experimental condition at 1-month, (beta=–1.15; SE 0.06; Cohen’s *d*=0.16; *P*=.01), 3-month (beta=–0.12; SE 0.05; Cohen’s *d*=0.09; *P*=.01), and 6-month (beta=–0.09; SE 0.05; Cohen’s *d*=0.03; *P*=.045) follow-up intervals (see Table 3 and Figure 6).

**Table 2:** Latent growth curve models presenting alcohol use intercepts and alcohol use slopes of intervention effects on alcohol use at 1-, 3-, and 6-month follow-up intervals (N=907).

Alcohol use intercepts and slopes at follow-up intervals		Weekly alcohol consumption		Binge drinking	
		Unstandardized estimate (SE)	*P*	Unstandardized estimate (SE)	*P*
**1 month (posttests 1-4)**					
	Baseline alcohol use on alcohol use intercept	0.86 (0.03)	<.001	0.71 (0.04)	<.001
	Baseline alcohol use on alcohol use slope	0.01 (0.02)	.60	–0.03 (0.02)	.14
	Intervention condition on alcohol use intercept	–2.70 (0.89)	.002	–0.21 (0.08)	.01
	Intervention condition on alcohol use slope	0.16 (0.44)	.73	0.04 (0.04)	.32
**3 months (posttests 1-12)**					
	Baseline alcohol use on alcohol use intercept	0.87 (0.03)	<.001	0.68 (0.03)	<.001
	Baseline alcohol use on alcohol use slope	–0.01 (0.003)	<.001	–0.01 (0.004)	.01
	Intervention condition on alcohol use intercept	–2.60 (0.73)	<.001	–0.15 (0.06)	.02
	Intervention condition on alcohol use slope	0.16 (0.09)	.08	0.01 (0.01)	.56
**6 months (posttests 1-25)**					
	Baseline alcohol use on alcohol use intercept	0.85 (0.03)	<.001	0.66 (0.03)	<.001
	Baseline alcohol use on alcohol use slope	–0.01 (0.001)	<.001	–0.004 (0.002)	.003
	Intervention condition on alcohol use intercept	–2.18 (0.65)	.001	–0.14 (0.05)	.01
	Intervention condition on alcohol use slope	0.08 (0.04)	.02	0.004 (0.003)	.19

**Table 3:** Intervention effects of alcohol use at 1-, 3-, and 6-month follow-ups by condition (intervention vs control): linear regression analyses unadjusted and adjusted for the outcome measures at baseline assessment (N=907).

Intervention effects at follow-up intervals		Group, mean (SD)		Beta (SE)	Cohen’s *d*	*P*
		Intervention (n=456)	Control (n=451)			
**Weekly alcohol consumption: unadjusted**						
	Baseline (4 pretests)	22.2 (12.9)	22.1 (13.8)			
	1 month (posttests 1-4)	24.0 (15.0)	26.5 (17.4)	–2.44 (1.09)	0.20	.03
	3 months (posttests 1-12)	23.1 (13.2)	24.9 (14.7)	–1.66 (0.94)	0.13	.08
	6 months (posttests 1-25)	22.9 (13.0)	24.0 (13.7)	–1.11 (0.89)	0.09	.21
**Weekly alcohol consumption: adjusted**						
	Baseline (4 pretests)	22.2 (12.9)	22.1 (13.8)			
	1 month (posttests 1-4)	24.0 (15.0)	26.5 (17.4)	–2.56 (0.74)	0.20	.001
	3 months (posttests 1-12)	23.1 (13.2)	24.9 (14.7)	–1.76 (0.60)	0.13	.003
	6 months (posttests 1-25)	22.9 (13.0)	24.0 (13.7)	–1.21 (0.58)	0.09	.04
**Binge drinking: unadjusted**						
	Baseline (4 pretests)	1.8 (1.0)	1.7 (1.1)			
	1 month (posttests 1-4)	1.9 (1.1)	2.0 (1.1)	–0.13 (0.08)	0.16	.08
	3 months (posttests 1-12)	1.8 (0.9)	1.9 (1.0)	–0.10 (0.06)	0.09	.12
	6 months (posttests 1-25)	1.8 (0.9)	1.8 (0.9)	–0.07 (0.25)	0.03	.25
**Binge drinking: adjusted**						
	Baseline (4 pretests)	1.8 (1.0)	1.7 (1.1)			
	1 month (posttests 1-4)	1.9 (1.1)	2.0 (1.1)	–0.15 (0.06)	0.16	.01
	3 months (posttests 1-12)	1.8 (0.9)	1.9 (1.0)	–0.12 (0.05)	0.09	.01
	6 months (posttests 1-25)	1.8 (0.9)	1.8 (0.9)	–0.09 (0.05)	0.03	.045

## Discussion

### Principal Results

This study is the first to test whether a Web-based brief alcohol intervention can sustain a reduction in alcohol use among heavy-drinking students at 1-, 3-, and 6-month follow-up intervals by means of an EMA approach with 25 posttests. The WDYD intervention did not reduce weekly alcohol consumption and frequency of binge drinking of participants in the experimental condition compared to participants in the control condition. Instead, the WDYD intervention was shown to be effective in preventing an increase in weekly alcohol consumption and frequency of binge drinking directly after the intervention that was sustained at 3 and 6 months postintervention. Ideally, participants in the experimental condition should reduce their alcohol use and participants in the control condition should stabilize after intervention exposure. However, these results revealed that participants in the experimental condition stabilized, whereas participants in the control condition deteriorated by increasing their alcohol use. Calendar-specific events might explain the increase in alcohol use that occurred among participants in the control condition from the beginning of February. The alcohol use patterns of participants in the control condition were similar to the patterns of binge drinking among freshmen that increased from winter break mid-December to New Year’s Eve, subsequently decreased up to the end of January and then increased again to the end of Spring Break in mid-March [34]. In the first week of February, participants in the experimental condition could benefit from the tips of the WDYD intervention to resist alcohol in different drinking situations. Exposure to the WDYD intervention might have led to an increase in drinking refusal self-efficacy, thereby making participants in the experimental condition less susceptible compared to participants in the control condition for calendar-specific events associated with elevated risk of excessive drinking [11]. In addition, binge drinking primarily occurs when students are with friends inside their homes, and outside their homes in bars, at parties, on dates, or during socializing activities [35]. It is reasonable to assume that students perceived the tips to resist alcohol as more relevant when they actually found themselves in drinking situations in which binge drinking occurs. This might explain the short-term preventive effect of the WDYD intervention for weekly alcohol consumption at 3 months postintervention and the long-term preventive effect of binge drinking frequency at 6 months postintervention.

In our previous study, we did not find significant main effects when we tested the effectiveness of the WDYD intervention at 1 and 6 months postintervention [5]. However, by using EMA and LGC modeling techniques, overall significant intervention effects were generated for weekly alcohol consumption and frequency of binge drinking that were sustained 3 and 6 months postintervention. This finding stresses the importance of using a large number of measurements in combination with appropriate statistical techniques to obtain higher precision in intervention effectiveness and minimize the danger of inaccurate conclusions about intervention effectiveness when using limited follow-up time points. Moreover, the use of EMA enables one to examine whether intervention effectiveness on the treatment outcome varied over time and helps determine the time at which the intervention effects stopped and the time at which booster sessions are needed to strengthen and/or extend intervention effects.

### Limitations and Strengths

Limitations of this study include the use of a large number of measurements by means of EMA, which might have affected the observed changes in the outcome measures by the act of assessing [36-38], yet participants in both conditions received weekly posttest measurements. If there was assessment reactivity, it could lead to underestimates of the true intervention effect [39]. Also, EMA could impose participant burden and reduce compliance because of the length of the survey entry, the frequency of responses, and the length of the study period [16]. Nonetheless, noncompliance and attrition were low in the current study. It seemed to be important to provide a briefing about the study procedure before the study onset, use short and well-conducted surveys, and offer a monetary incentive after study completion. In addition, the use of EMA might even alleviate sample size requirements because it provides more refined outcome measures that are more sensitive to change, thereby making studies less difficult and less expensive to conduct [16]. Additionally, the effect sizes of the WDYD intervention were small but comparable to those reported in other Web-based brief alcohol interventions [40,41]. Despite the small absolute differences in alcohol use between the conditions, the advantage lies in the inclusion of all the effects of the WDYD intervention over time across a far larger group of heavy drinkers with less serious alcohol-related problems resulting in a greater societal gain than reducing problem drinking among a smaller number of dependent drinkers, known as the prevention paradox, that is used to justify a population strategy of prevention [42]. Further, the representativeness of the study sample might have been affected because of the convenience sampling strategy, although the majority of trials on Web-based brief alcohol interventions have used this type of sampling strategy (eg, [43]) in which participants are selected based on availability. Moreover, contamination between conditions might have occurred if participants in the experimental condition shared the link of the WDYD intervention with participants in the control condition. Nonetheless, WDYD is not yet available online; thereby, it reduces the likelihood of contamination between conditions. Additionally, the EMA measurements relied on self-reported measures with 7-day recall, which still remains subject to measurement errors because data were not collected in the event and precise recall of alcohol use decreases after 2 or 3 days due to memory deficits [10]. True in-the-event measures would be very difficult over long time periods. However, this is the first study using 30 weekly EMA measurements to assess outcome measures, thereby generating outcome measures that are much closer to the actual drinking behavior of individuals than any other trial on Web-based brief alcohol interventions in the current alcohol prevention literature. Another limitation is that the outcome measures of weekly alcohol consumption and frequency of binge drinking have not been validated for online use. However, these outcome measures have been validated in paper-based surveys [27,44]. In addition, research has shown that online survey data can be equal or superior to that of equivalent paper-based survey data [45]. Furthermore, participants were not blinded to the assigned interventions, which is a common limitation in Web-based trials [46]. Participants who are aware that they have been assigned to the experimental condition might have favorable expectations or increased apprehension and participants assigned to the control condition might feel deprived or relieved, which can affect their responses on the outcome measures. Finally, one should be careful in generalizing our findings to students who are not ready to change their alcohol use, individuals younger than 18 years, and those who do not attend or have not attended a college or university.

Strengths of this study included the online weekly EMA methodology for assessing alcohol use and intervention effectiveness over time while maintaining a high retention rate. First, the use of a large number of measurements over time by means of EMA enabled us to assess changes in alcohol use and intervention effectiveness over time while taking into account the fluctuating nature of alcohol use among students. Second, the coverage strategy of EMA minimized recall bias because of a relative short reference period (ie, 1 week), thereby generating more ecologically valid outcome measures of self-reported drinking behaviors [16]. Third, the use of online surveys had the advantage over paper-and-pencil surveys because it reduced the likelihood of entry errors while improving cost-effectiveness [47]. Fourth, the use of EMA in combination with LGC modeling techniques allowed diminishing statistical errors by generating overall intervention effects resulting in more reliable outcome measures and a higher precision in intervention effectiveness. In addition, the WDYD intervention is based upon the intervention mapping protocol, which is a sound framework for theoretical- and evidence-based development, implementation, and evaluation of effective behavior change interventions [13]. Moreover, the WDYD intervention incorporated components (eg, personalized normative feedback) that are successful in reducing heavy drinking among student populations [48].

### Future Directions

The findings of the current study suggest that the WDYD intervention can prevent an increase in weekly alcohol consumption and frequency of binge drinking among heavy-drinking students that is sustained at 3 to 6 months postintervention. The collaboration with the Trimbos Institute can ensure an adequate large-scale implementation of the WDYD intervention by incorporating it in their materials and programs [12]. In addition, the findings indicate the relevance of including a large number of measurements by means of EMA for assessing the outcome measures and evaluating the intervention effectiveness to obtain higher precision in future alcohol prevention trials. If a large number of measurements with extremely short reference periods (ie, 2 hours) are used to assess outcome measures, smartphones might be more beneficial than online surveys because they can capture data regardless of time and location of the participant [49]. Moreover, future research should identify whether alcohol-related cognitions (eg, self-efficacy) account for the observed outcomes to help explain why Web-based brief alcohol interventions are effective in reducing or, in our case, preventing an increase in alcohol use among heavy-drinking students, especially considering that most Web-based brief alcohol interventions are designed to affect alcohol-related cognitions that determine heavy drinking in young adults [50,51].

### Conclusions

The WDYD Web-based brief alcohol intervention was shown to be effective in preventing an increase in weekly alcohol consumption and frequency of binge drinking directly after the intervention among heavy-drinking students that was sustained at 3 and 6 months postintervention. Moreover, the findings emphasize the strengths of using EMA and statistical techniques, such as LGC, in testing the intervention effectiveness that would otherwise remain undetected.

## Multimedia Appendix 1

CONSORT-EHEALTH checklist V1.6.2 [46].

## Abbreviations

AUDIT: Alcohol Use Disorders Identification Test
CFI: Comparative Fit Index
CONSORT: Consolidated Standards of Reporting Trials
EMA: ecological momentary assessment
ESM: experience sampling methods
ITT: intent-to-treat
LGC: latent growth curve
RMSEA: Root Mean Square Error of Approximation
TLI: Tucker-Lewis Index
WDYD: What Do You Drink
