# Supplementary material for: Using Ecological Momentary Assessment to Test the Effectiveness of a Web-Based Brief Alcohol Intervention Over Time Among Heavy-Drinking Students: Randomized Controlled Trial
Source: J Med Internet Res. 2014 Jan 8;16(1):e5. doi: 10.2196/jmir.2817 (PMC3906701; doi:10.2196/jmir.2817)
Supplement: Supplementary file 1 [file jmir_v16i1e5_app1.pdf]

CONSORT-EHEALTH Checklist V1.6.2 Report

(based on CONSORT-EHEALTH V1.6), available at [http://tinyurl.com/consort-ehealth-v1-6].

2817

Date completed

7/4/2013 11:12:36

by

Carmen Voogt

"Using Ecological Momentary Assessment to test the Effectiveness of the Web-based Brief Alcohol Intervention ‘What Do You Drink’ over Time among Heavy Drinking Students: A 2-arm Parallel Group Randomized Controlled Trial"

TITLE

1a-i) Identify the mode of delivery in the title

Yes: "Using Ecological Momentary Assessment to test the Effectiveness of the Web-based Brief Alcohol Intervention ‘What Do You Drink’ over Time among Heavy Drinking Students: A 2-arm Parallel Group Randomized Controlled Trial"

1a-ii) Non-web-based components or important co-interventions in title

Not applicable: there were no non-web-based components or important co-interventions.

1a-iii) Primary condition or target group in the title

Yes: "Using Ecological Momentary Assessment to test the Effectiveness of the Web-based Brief Alcohol Intervention ‘What Do You Drink’ over Time among Heavy Drinking Students: A 2-arm Parallel Group Randomized Controlled Trial"

ABSTRACT

1b-i) Key features/functionalities/components of the intervention and comparator in the METHODS section of the ABSTRACT

Yes, although partly. The inclusion of theories and principles used for designing the WDYD intervention in the Methods section of the Abstract is too extensive since words are limited to a maximum of 450. This information is provided in the Methods section under Interventions. In the Methods section of the Abstract we mentioned that: "A total of 907 participants were randomized into the experimental (n = 456: single-session, fully-automated, and freely accessible WDYD intervention) or control condition (n = 451: no intervention)."

1b-ii) Level of human involvement in the METHODS section of the ABSTRACT

Yes: "A total of 907 participants were randomized into the experimental (n = 456: single-session, fully-automated, and freely accessible WDYD intervention) or control condition (n = 451: no intervention)."

1b-iii) Open vs. closed, web-based (self-assessment) vs. face-to-face assessments in the METHODS section of the ABSTRACT

Yes: "A purely web-based 2-arm parallel group randomized controlled trial applying an ecological momentary assessment approach with 30 data time-points was conducted in the Netherlands (2010-2011). Participants were recruited offline and online. A total of 907 participants were randomized into the experimental (n = 456: single-session, fully-automated, and freely accessible WDYD intervention) or control condition (n = 451: no intervention). Weekly alcohol consumption and frequency of binge drinking were self-assessed outcome measures."

1b-iv) RESULTS section in abstract must contain use data

Yes. In the Methods section of the Abstract it is stated that "A total of 907 participants were randomized into the experimental (n = 456: single-session, fully-automated, and freely accessible WDYD intervention) or control condition (n = 451: no intervention). In the Results section of the Abstract it is stated that "Attrition rates of 907 participants were 110 (12.1%), 130 (14.3%), and 162 (17.9%) at 1, 3, and 6 six months follow-up."

1b-v) CONCLUSIONS/DISCUSSION in abstract for negative trials

Not applicable: this is a positive trial (primary and secondary outcome measures were changed).

INTRODUCTION

2a-i) Problem and the type of system/solution

Yes: “Given the high prevalence and social and economic costs attributable to heavy drinking among young adults, there is an urgent need for adequate interventions. The widespread growth and availability of computer technology and the Internet has provided the opportunity to deliver interventions via the web, which are especially advantageous for young adults since it allows them to access information at a self-selected time and place while remaining anonymous.” Therefore, the single-session, fully-automated, and freely accessible WDYD intervention was developed that "aims to detect and reduce heavy drinking of young adults."

2a-ii) Scientific background, rationale: What is known about the (type of) system

Yes: "Web-based brief alcohol interventions are found to be effective in reducing alcohol use among students when measured at few follow-up time-points. To date, no studies tested web-based brief alcohol intervention effectiveness over time by using multiple time-points. Therefore, this study tested “whether the What Do You Drink (WDYD) web-based brief alcohol intervention can sustain a reduction in alcohol use among heavy drinking students aged 18-24 years after 1, 3, and 6 months follow-up.”

METHODS

3a) CONSORT: Description of trial design (such as parallel, factorial) including allocation ratio

Yes: "To our knowledge, this is the first study to test whether a web-based brief alcohol intervention can sustain a reduction in alcohol use among heavy drinking students after 1, 3, and 6 months follow-up. Therefore, we used an online weekly EMA approach with 26 time-points and conducted LGC analyses to model individual change in weekly alcohol consumption and frequency of binge drinking over 3 time-periods by condition. By aggregating the means of outcome measures across multiple time-points using EMA, we hypothesized that participants in the experimental condition would reduce their alcohol use (intercept) compared to participants in the control condition directly after exposure to the WDYD intervention. Based on web-based brief alcohol interventions that have produced long term effects, it was hypothesized that the reduction would sustain (slope) after 6 months follow-up.”

3b) CONSORT: Important changes to methods after trial commencement (such as eligibility criteria), with reasons

Not applicable: there were no important changes to methods after trial commencement.

3b-i) Bug fixes, Downtimes, Content Changes

Not applicable: there were no bug fixes, downtimes, or content changes.

4a) CONSORT: Eligibility criteria for participants

Yes: "To be included in the study, students had to 1) be between 18 to 24 years of age, 2) report heavy drinking in the past 6 months, 3) be ready to change their alcohol use, 4) have daily access to the Internet (and be literate), and 5) sign an online informed consent form."

4a-i) Computer / Internet literacy

Yes: "To be included in the study, students had to 1) be between 18 to 24 years of age, 2) report heavy drinking in the past 6 months, 3) be ready to change their alcohol use, 4) have daily access to the Internet (and be literate), and 5) sign an online informed consent form."

4a-ii) Open vs. closed, web-based vs. face-to-face assessments:

Yes: "A convenience sampling strategy was used to recruit heavy drinking students offline by distributing flyers at Higher Professional Education (HBO) institutions and universities and online by sending e-mails with information about the study from September until December 2010." Moreover, it is stated that the "trial was purely web-based since there were no face-to-face components in the intervention and for assessing the outcomes measures."

4a-iii) Information giving during recruitment

Yes: "The cover story was that students had to evaluate newly developed health education materials addressing alcohol use and that they had to judge these materials in order to reduce the risk of social desirability bias. Students were blinded to the aim of the study until the end of the EMA-study."

4b) CONSORT: Settings and locations where the data were collected

Yes: "In total, 30 EMA-measurements were conducted online from January until August in 2011 to assess outcomes measures with 4 pre-tests and 26 post-tests. After 4 pre-tests in January, participants in the experimental condition were exposed to the WDYD intervention while those in the control condition received no intervention. Directly after intervention exposure in the first week of February, participants in both conditions received the first post-test. One week after the intervention, all participants received weekly EMA post-test measurements for 6 months from February until August. EMA-measurements were taken on Monday mornings. All participants received e-mail with the instructions on the use of the survey, and they were asked to respond to the survey before midnight. Each survey took about 10 minutes to complete and contained identical questions about participants' weekly alcohol consumption, frequency of binge drinking, and drinking refusal self-efficacy. In addition, extended surveys were administered at baseline, immediately after the intervention, 1 month follow-up, and 6 months follow-up. These extended surveys additionally included questions concerning alcohol related cognitions, cost-effectiveness, and problem drinking. Completion time of the extended surveys was roughly 20 minutes. Paper and pencil surveys with identical content were provided to participants in case they were unable to access the Internet. Participants who failed to complete the survey on Mondays received a short text message on their mobile phones on Tuesdays to remind them. Those who still did not complete the survey on Tuesdays were reminded by a telephone call on Wednesdays."

4b-i) Report if outcomes were (self-)assessed through online questionnaires

Yes: "Weekly alcohol consumption and frequency of binge drinking were self-assessed outcome measures through online surveys that have not been validated for online use."

4b-ii) Report how institutional affiliations are displayed

Not applicable.

5) CONSORT: Describe the interventions for each group with sufficient details to allow replication, including how and when they were actually administered

5-i) Mention names, credential, affiliations of the developers, sponsors, and owners

Yes: "The single-session, fully-automated, and freely accessible WDYD intervention was developed in collaboration with the Trimbos Institute (Netherlands Institute of Mental Health and Addiction) by using the Intervention Mapping protocol."

5-ii) Describe the history/development process

Yes: "The single-session, fully-automated, and freely accessible WDYD intervention was developed in collaboration with the Trimbos Institute (Netherlands Institute of Mental Health and Addiction) by using the Intervention Mapping protocol. Content is based on Motivational Interviewing principles and parts of the I-Change model in which knowledge, social norms, and self-efficacy are embedded as the most changeable determinants of behaviour change." A full description of the development process of the WDYD intervention is given in another paper as states like this "A full description of the WDYD intervention is given elsewhere."

5-iii) Revisions and updating

Not applicable: the WDYD intervention unwent no major changes during the evaluation process.

5-iv) Quality assurance methods

Not applicable.

5-v) Ensure replicability by publishing the source code, and/or providing screenshots/screen-capture video, and/or providing flowcharts of the algorithms used

Yes: Screenshots of the WDYD intervention are provided as Figure 1 ("Screenshots of the What Do You Drink intervention part 1") and Figure 2 ("Screenshots of the What Do You Drink intervention part 2").

5-vi) Digital preservation

No: The WDYD intervention is not yet available online under the final URL.

5-vii) Access

Yes: The WDYD intervention was freely accessible. "In total, 30 EMA-measurements were conducted online from January until August in 2011 to assess outcomes measures with 4 pre-tests and 26 post-tests. After 4 pre-tests in January, participants in the experimental condition were exposed to the WDYD intervention while those in the control condition received no intervention. Directly after intervention exposure in the first week of February, participants in both conditions received the first post-test. One week after the intervention, all participants received weekly EMA post-test measurements for 6 months from February until August."

5-viii) Mode of delivery, features/functionalities/components of the intervention and comparator, and the theoretical framework

Yes: "The single-session, fully-automated, and freely accessible WDYD intervention was developed in collaboration with the Trimbos Institute (Netherlands Institute of Mental Health and Addiction) by using the Intervention Mapping protocol [16]. Content is based on Motivational Interviewing principles and parts of the I-Change model in which knowledge, social norms, and self-efficacy are embedded as the most changeable determinants of behaviour change". A full description of the development process of the WDYD intervention is given in another paper as states like this "A full description of the WDYD intervention is given elsewhere."

"The first part of the WDYD intervention focuses on increasing the users' awareness of the potential problems, consequences, and risks associated with their drinking behaviour. It contains a homepage and a screening test with personalized feedback that was delivered in a non-judgmental, non-confrontational, and non-aversive way. The screening test included participants' self-reported name, sex, age, education level, weight, alcohol use, readiness to change alcohol use, average expenses on consumed alcohol beverages, and descriptive social norms. Personalized feedback consisted of advice about drinking according to low-risk drinking guidelines, personal drinking profile (quantity-frequency consumed in last year), estimates of calorie intake, increases in weight, money expenses due to drinking, and a comparison of personal use rates with the national norms of same-sex peers to correct misperceptions of descriptive social norms. The personalized feedback was based on the individuals' personal situation implying that the WDYD intervention is "tailored". The second part of WDYD focuses on setting and maintaining drinking goals and strengthening users' drinking refusal self-efficacy to succeed and maintain drinking goals by providing tips to resist alcohol in different drinking situations. Participants were able to track their progress through the WDYD intervention, which took approximately 20 minutes to complete."

5-ix) Describe use parameters

Yes: The WDYD intervention was a single session intervention that took about 20 monutes to complete.

"The single-session, fully-automated, and freely accessible WDYD intervention was developed in collaboration with the Trimbos Institute (Netherlands Institute of Mental Health and Addiction) by using the Intervention Mapping protocol."

"Participants were able to track their progress through the WDYD intervention, which took approximately 20 minutes to complete"

5-x) Clarify the level of human involvement

Yes: "This trial was purely web-based since there were no face-to-face components in the intervention and for assessing the outcomes measures."

5-xi) Report any prompts/reminders used

Yes: No reminders were used to use the application since the WDYD intervention was a single session character. Reminders were only used for assessing the outcomes measures. "Participants who failed to complete the survey on Mondays received a short text message on their mobile phones on Tuesdays to remind them. Those who still did not complete the survey on Tuesdays were reminded by a telephone call on Wednesdays.".

5-xii) Describe any co-interventions (incl. training/support)
Not applicable: there are no co-interventions.
6a) CONSORT: Completely defined pre-specified primary and secondary outcome measures, including how and when they were assessed
Yes: "The primary outcome measure was weekly alcohol consumption after 1, 3, and 6 months follow-up. The Dutch version of the Alcohol Weekly Recall was used to assess the mean number of glasses of standard alcohol units consumed in the previous 7 days. Participants were asked to indicate retrospectively the exact number, size and type of alcohol beverage they consumed on each day in the previous 7 days. An overview of standard units for various beverages was provided to guarantee standardized responses. In total, 1.47% of the participants scored 3 standard deviations above the sample mean of weekly alcohol consumption and were given that value in order to retain outliers in the analyses (resulting range 0 to 109)."
"The secondary outcome measures were frequency of binge drinking after 1, 3, and 6 months follow-up and 30 EMA-measurements on weekly alcohol consumption and frequency of binge drinking. Binge drinking frequency was defined as the number of days in the previous week on which participants had drunk 5 or more glasses of standard alcohol units per occasion. The frequency of binge drinking was measured on an 8-point Likert scale ranging from (0) “never” to (7) “every day”."

6a-i) Online questionnaires: describe if they were validated for online use and apply CHERRIES items to describe how the questionnaires were designed/deployed
Yes: "Weekly alcohol consumption and frequency of binge drinking were self-assessed outcome measures through online surveys that have not been validated for online use."
6a-ii) Describe whether and how “use” (including intensity of use/dosage) was defined/measured/monitored
No.
6a-iii) Describe whether, how, and when qualitative feedback from participants was obtained
No.
6b) CONSORT: Any changes to trial outcomes after the trial commenced, with reasons
Not applicable: there were no changes to trial outcomes after the trial commenced.
7a) CONSORT: How sample size was determined
7a-i) Describe whether and how expected attrition was taken into account when calculating the sample size
Yes: “To detect an increase in the percentage of participants showing low-risk drinking guidelines after 1 month of 42% in the experimental condition versus 31% in the control condition with a 2-sided 5% significance level and a power of 80%, a sample size of 908 participants was necessary given an anticipated dropout rate of 30% after randomization.”
7b) CONSORT: When applicable, explanation of any interim analyses and stopping guidelines
Not applicable.
8a) CONSORT: Method used to generate the random allocation sequence
Not applicable.
8b) CONSORT: Type of randomisation; details of any restriction (such as blocking and block size)
Yes: “Students who met the inclusion criteria were randomly assigned to the WDYD intervention condition (n = 456) or to the control condition (n = 451) in blocks of 4 using a computerized random number generator by an independent researcher of the Behavioural Science Institute that could not influence or predict the randomization result. Participants were not blinded to randomization results. Randomization was stratified by sex and educational level before the baseline assessment in January 2011.”
9) CONSORT: Mechanism used to implement the random allocation sequence (such as sequentially numbered containers), describing any steps taken to conceal the sequence until interventions were assigned
Yes: “Students who met the inclusion criteria were randomly assigned to the WDYD intervention condition (n = 456) or to the control condition (n = 451) in blocks of 4 using a computerized random number generator by an independent researcher of the Behavioural Science Institute that could not influence or predict the randomization result. Participants were not blinded to randomization results. Randomization was stratified by sex and educational level before the baseline assessment in January 2011.”
10) CONSORT: Who generated the random allocation sequence, who enrolled participants, and who assigned participants to interventions
Yes: “Students who met the inclusion criteria were randomly assigned to the WDYD intervention condition (n = 456) or to the control condition (n = 451) in blocks of 4 using a computerized random number generator by an independent researcher of the Behavioural Science Institute that could not influence or predict the randomization result. Participants were not blinded to randomization results. Randomization was stratified by sex and educational level before the baseline assessment in January 2011.”
11a) CONSORT: Blinding - If done, who was blinded after assignment to interventions (for example, participants, care providers, those assessing outcomes) and how
11a-i) Specify who was blinded, and who wasn’t
Yes: "Furthermore, participants were not blinded to the assigned interventions, which is a common limitation in web-based trials."
11a-ii) Discuss e.g., whether participants knew which intervention was the “intervention of interest” and which one was the “comparator”
Yes: "Participants were not blinded to randomization results."
11b) CONSORT: If relevant, description of the similarity of interventions
Not applicable.
12a) CONSORT: Statistical methods used to compare groups for primary and secondary outcomes
Yes: “Second, LGC analyses were conducted to model individual change in alcohol use over time by condition as reported after 1, 3, and 6 months follow-up. LGC analyses were conducted over 3 time-periods to limit the chance of reporting outlier trajectories. The models without condition status and baseline levels of alcohol use were tested first. Subsequently, the growth curves were regressed on condition status for weekly alcohol consumption and frequency of binge drinking separately while adjusting for baseline levels of alcohol use. A random-effect parameter for educational institutions was not included in the models since variation in participants between institutions was expected to be limited since all participants needed to meet the inclusion criteria of the study. Unstandardised intercepts (B0), representing alcohol use directly after the intervention, and unstandardised slopes (B1), representing the change of alcohol use over time, were reported. Global fit indices were used to assess model fit for each construct: chi-square statistic, Comparative Fit Index (CFI), Tucker-Lewis fit Index (TLI), and Root Mean Square Error of Approximation (RMSEA). In parallel with the LGC analyses, linear regression analyses were conducted for weekly alcohol consumption and frequency of binge drinking at 1, 3, and 6 months follow-up and presented as unadjusted and adjusted for baseline levels of alcohol use. For the linear regression analyses, unstandardized coefficients (Betas), standard errors (SE), and Cohen’s d (i.e., M1 – M2 / √(SD12 + SD 22) / 2) effect sizes were provided.”
12a-i) Imputation techniques to deal with attrition / missing values

Yes: "Data were analyzed according the intent-to-treat (ITT) principle. Missing data were handled employing multiple imputations using the predictive mean matching method (MMS). Twenty imputed datasets were evaluated with P < .05 as criterion for statistical significance by averaging the results (i. e., pooling)."

12b) CONSORT: Methods for additional analyses, such as subgroup analyses and adjusted analyses

Not applicable.

RESULTS

13a) CONSORT: For each group, the numbers of participants who were randomly assigned, received intended treatment, and were analysed for the primary outcome

Yes: "Figure 3 illustrates the participant flow through the study and the data collection with 30 EMA-measurements. Originally, 913 students were included in the study. However, 6 students did not fill in the baseline assessment and were consequently excluded from the study. Finally, 907 participants were enrolled in the EMA study, randomized into the experimental condition (n = 456 (50.3%)) or control condition (n = 451 (49.7%)), and eligible for the intention-to-treat analyses. In total, 745 (82.1%) completed the baseline assessment and all 25 EMA follow-ups."

13b) CONSORT: For each group, losses and exclusions after randomisation, together with reasons

Yes: "Due to lack of interest and time, attrition rates were 12.1% (n = 110, 62 in the intervention condition and 48 in the control condition), 14.3% (n = 130, 72 in the intervention condition and 58 in the control condition), and 17.9% (n = 162, 87 in the intervention condition and 75 in the control condition) at 1, 3, and 6 six months follow-up, respectively."

13b-i) Attrition diagram

No, but a participant flow through the study and the data collection with 30 EMA-measurements is provided as Figure 3.

14a) CONSORT: Dates defining the periods of recruitment and follow-up

Yes: “A convenience sampling strategy was used to recruit heavy drinking students offline by distributing flyers at Higher Professional Education (HBO) institutions and universities and online by sending e-mails with information about the study from September until December 2010.”

“In total, 30 EMA-measurements were conducted online from January until August in 2011 to assess outcomes measures with 4 pre-tests and 26 post-tests. After 4 pre-tests in January, participants in the experimental condition were exposed to the WDYD intervention while those in the control condition received no intervention. Directly after intervention exposure in the first week of February, participants in both conditions received the first post-test. One week after the intervention, all participants received weekly EMA post-test measurements for 6 months from February until August.”

14a-i) Indicate if critical “secular events” fell into the study period

Not applicable.

14b) CONSORT: Why the trial ended or was stopped (early)

Not applicable.

15) CONSORT: A table showing baseline demographic and clinical characteristics for each group

Yes: Table 1 provides baseline demographic characteristics.

15-i) Report demographics associated with digital divide issues

Yes: "Of the 907 participants, 547 (60.3%) were male, 667 (73.5%) attended university, and 194 (21.4%) were ready to reduce alcohol use in the near future. The screening survey was administered between September and December 2010, whereas the baseline assessment was administered in January 2011, which might explain the lower rates of participant’s readiness to change alcohol use at baseline. On average, participants were 20.8 (SD = 1.7) years old. At baseline, participants reported to consume 21.9 (SD = 13.5) alcohol units per week and reported to have 1.8 (1.0) occasions in which they drunk 5 or more glasses of alcohol units on average per week (see Table 1). No significant differences (P > .05) emerged between conditions in demographic characteristics and outcome measures at baseline (analyses not shown here)."

16a) CONSORT: For each group, number of participants (denominator) included in each analysis and whether the analysis was by original assigned groups

16-i) Report multiple “denominators” and provide definitions

Yes: Data were analyzed according the intent-to-treat (ITT) principle and conducted among 907 participants.

16-ii) Primary analysis should be intent-to-treat

Yes: "Data were analyzed according the intent-to-treat (ITT) principle. Missing data were handled employing multiple imputations using the predictive mean matching method (MMS) [32]. Twenty imputed datasets were evaluated with P < .05 as criterion for statistical significance by averaging the results (i.e., pooling)."

17a) CONSORT: For each primary and secondary outcome, results for each group, and the estimated effect size and its precision (such as 95% confidence interval)

Yes: "The weekly alcohol consumption model fitted the observed data sufficiently at follow-up assessments, except for the RMSEA at 1 month follow-up. LGM analyses revealed that participants in the experimental condition had a significantly lower weekly alcohol consumption compared to participants in the control condition directly after the intervention. The intercept difference in alcohol units between conditions sustained after 3 months follow-up (B0 = -2.60, P < .001; B1 = 0.16, P = .08), but fades out over time resulting in a significant slope of the latent growth curve after 6 months follow-up (B0 = -2.18, P = .001; B1 = 0.08, P = .02). Linear regression analyses indicated that the intercept difference resulted from a significantly higher increase in alcohol units per week for participants in the control condition compared to those in the experimental condition at 1, (Beta = -2.56; SE = 0.74; Cohen’s d = 0.20; P = .001), 3 (Beta = -1.76; SE = 0.60; Cohen’s d = 0.13; P = .003), and 6 (Beta = -1.21; SE = 0.58; Cohen’s d = 0.09; P = .04) months follow-up."

"The frequency of binge drinking model provided reasonable fit to the data at all 3 follow-up assessments. According to the LGM analyses, the average number of occasions in the previous week on which participants in the experimental condition drunk 5 or more glasses of alcohol was significantly lower compared to participants in the control condition. The intercept difference in frequency of binge drinking sustained after 6 months follow-up (B0 = -0.14, P = .01; B1 = 0.00, P = .19) and resulted from a larger increase in this outcome for participants in the control condition relative to participants in the experimental condition at 1, (Beta = -1.15; SE = 0.06; Cohen’s d = 0.16; P = .01), 3 (Beta = -0.12; SE = 0.05; Cohen’s d = 0.09; P = .01), and 6 (Beta = -0.09; SE = 0.05; Cohen’s d = 0.03; P = .045) months follow-up."

17a-i) Presentation of process outcomes such as metrics of use and intensity of use

No.

17b) CONSORT: For binary outcomes, presentation of both absolute and relative effect sizes is recommended

Not applicable.

18) CONSORT: Results of any other analyses performed, including subgroup analyses and adjusted analyses, distinguishing pre-specified from exploratory

Not applicable.

18-i) Subgroup analysis of comparing only users

Not applicable.

19) CONSORT: All important harms or unintended effects in each group

Not applicable.

19-i) Include privacy breaches, technical problems

Not applicable.

19-ii) Include qualitative feedback from participants or observations from staff/researchers

No.

DISCUSSION

20) CONSORT: Trial limitations, addressing sources of potential bias, imprecision, multiplicity of analyses

20-i) Typical limitations in ehealth trials

Yes: "Limitations of this study included that the use of multiple time-points by means of EMA might have affected the observed changes in the outcomes by the act of assessing, yet participants in both conditions received weekly post-test measurements. If there was assessment reactivity, it could lead to underestimates of the true intervention effect. Also, EMA could impose participant burden and reduce compliance due to the length of the survey entry, the frequency of responses, and the length of the study period. Nonetheless, noncompliance and attrition were low in the current study. It seemed to be important to provide a briefing about the study procedure before the study onset, use short and well-conducted surveys, and offer a monetary incentive after study completion. Besides, the use of EMA might even alleviate sample size requirements since it provides more refined outcomes that are more sensitive to change, thereby making studies less difficult and less expensive to conduct. Additionally, the effects sizes of the WDYD intervention were small but comparable to those reported in other web-based brief alcohol intervention. Despite the small absolute differences in alcohol use between the conditions, the advantage lies in the aggregation of the effects of the WDYD intervention over time across a far larger group of heavy drinkers with less serious alcohol-related problems resulting in a greater societal gain than reducing problem drinking amongst a smaller number of dependent drinkers, known as the “prevention paradox” that is being used to justify a population strategy of prevention. Further, the representativeness of the study sample might have been affected due to the convenience sampling strategy, although the majority of trials on web-based brief alcohol interventions have used this type of sampling strategy in which participants are selected based on availability. Moreover, contamination between conditions might have occurred if participants in the experimental condition shared the link of the WDYD intervention with participants in the control condition. Nonetheless, WDYD is not yet available online; thereby, it reduces the likelihood of contamination between conditions. Additionally, the EMA-measurements relied on self-reported measures with 7 days recall, which still remains subject to measurement errors since data were not collected at the moment and precise recall of alcohol use decreases after 2 or 3 days due to memory deficits. Furthermore, participants were not blinded to the assigned interventions, which is a common limitation in web-based trials. Participants who are aware that they have been assigned to the experimental condition might have favorable expectations or increased apprehension and participants assigned to the control condition might feel deprived or relieved that can affect their responses on the outcome measures. Finally, one should be careful in generalizing our findings to individuals younger than 18 and those who have not attended a college or university."

21) CONSORT: Generalisability (external validity, applicability) of the trial findings

21-i) Generalizability to other populations

Yes: "Finally, one should be careful in generalizing our findings to individuals younger than 18 and those who have not attended a college or university."

21-ii) Discuss if there were elements in the RCT that would be different in a routine application setting

Yes: "First, the use of multiple follow-ups over time by means of EMA enabled to assess changes in alcohol use and intervention effectiveness over time while taking into account the fluctuating nature of alcohol use among students. Second, the coverage strategy of EMA minimized recall bias due to a relative short reference period (i.e., 1 week), thereby generating more ecologically valid outcome measures of self-reported drinking behaviours."

22) CONSORT: Interpretation consistent with results, balancing benefits and harms, and considering other relevant evidence

22-i) Restate study questions and summarize the answers suggested by the data, starting with primary outcomes and process outcomes (use)

Yes: "This study is the first to test whether a web-based brief alcohol intervention can sustain a reduction in alcohol use among heavy drinking students after 1, 3, and 6 months follow-up by means of an EMA approach with 25 follow-ups. Unexpectedly, the WDYD intervention did not reduce weekly alcohol consumption and frequency of binge drinking of participants in the experimental condition compared to participants in the control condition. Instead, the intervention prevented an increase in weekly alcohol consumption and frequency of binge drinking directly after the intervention that sustained after 3 and 6 months post-intervention, respectively."

22-ii) Highlight unanswered new questions, suggest future research

Yes: "Moreover, future research should identify whether alcohol related cognitions (e.g., self-efficacy) account for the observed outcomes to help explain why web-based brief alcohol interventions are effective in reducing or, in our case, preventing an increase in alcohol use among heavy drinking students, especially considering that most web-based brief alcohol interventions are designed to affect alcohol related cognitions that determine heavy drinking in young adults."

Other information

23) CONSORT: Registration number and name of trial registry

Yes: "Trial Registration: Netherlands Trial Register NTR2665."

24) CONSORT: Where the full trial protocol can be accessed, if available

Yes: The full trial can be assessed at: http://www.biomedcentral.com/1471-2458/11/231 (Voogt CV, Poelen EAP, Kleinjan M, Lemmers LACJ, Engels RCME. Targeting young drinkers online: The effectiveness of a web-based brief alcohol intervention in reducing heavy drinking among college students: Study protocol of a two-arm parallel group randomized controlled trial. BMC Public Health 2011.)

25) CONSORT: Sources of funding and other support (such as supply of drugs), role of funders

Yes: "The major funding agency ZonMw, The Netherlands Organization for Health Research and Development, supported this study (grant number 50-50110-96-682)."

X26-i) Comment on ethics committee approval

Yes: "Ethical approval was provided by the Ethical Committee of the Faculty of Social Sciences at Radboud University Nijmegen."

x26-ii) Outline informed consent procedures

Yes: "To be included in the study, students had to 1) be between 18 to 24 years of age, 2) report heavy drinking in the past 6 months, 3) be ready to change their alcohol use, 4) have daily access to the Internet (and be literate), and 5) sign an online informed consent form."

X26-iii) Safety and security procedures

No.

X27-i) State the relation of the study team towards the system being evaluated

Yes: "Conflict of Interest: None declared."
